# Quantifying pulse oximeter accuracy during hypoxemia and severe anemia using an in vitro circulation system

**DOI:** 10.1007/s10877-023-01031-3

**Published:** 2023-06-02

**Authors:** Raymond Gylys, John Feiner, Jonas Pologe, Theodore Delianides, Stephanie Sutter, Philip Bickler, Michael S. Lipnick

**Affiliations:** 1 Hypoxia Lab, Department of Anesthesia, University of California San Francisco, San Francisco, CA USA; 2 Kestrel Labs, Inc., Boulder, CO USA; 3 Center for Health Equity in Surgery and Anesthesia, University of California San Francisco, San Francisco, CA USA

**Keywords:** Monitoring, Pulse oximetry, Accuracy, In vitro circulation system, Severe anemia, Hypoxemia

## Abstract

Anemia and hypoxemia are common clinical conditions that are difficult to study and may impact pulse oximeter performance. Utilizing an in vitro circulation system, we studied performance of three pulse oximeters during hypoxemia and severe anemia. Three oximeters including one benchtop, one handheld, and one fingertip device were selected to reflect a range of cost and device types. Human blood was diluted to generate four hematocrit levels (40%, 30%, 20%, and 10%). Oxygen and nitrogen were bubbled through the blood to generate a range of oxygen saturations (O_2_Hb) and the blood was cycled through the in vitro circulation system. Pulse oximeter saturations (SpO_2_) were paired with simultaneously-measured O_2_Hb readings from a reference CO-oximeter. Data for each hematocrit level and each device were least-squares fit to a 2nd-order equation with quality of each curve fit evaluated using standard error of the estimate. Bias and average root mean square error were calculated after correcting for the calibration difference between human and in vitro circulation system calibration. The benchtop oximeter maintained good accuracy at all but the most extreme level of anemia. The handheld device was not as accurate as the benchtop, and inaccuracies increased at lower hematocrit levels. The fingertip device was the least accurate of the three oximeters. Pulse oximeter performance is impacted by severe anemia in vitro. The use of in vitro calibration systems may play an important role in augmenting in vivo performance studies evaluating pulse oximeter performance in challenging conditions.

## Introduction

Anemia and hypoxemia are common clinical conditions yet relatively little is known about the performance of pulse oximeters in severely anemic patients. In this study, we utilized an in vitro circulation system (IVCS) to study the performance of three pulse oximeters ranging in price from $20 to > $1000 during hypoxemia and severe anemia.

Pulse oximetry is universally recognized as an essential tool for safe clinical care, especially in the perioperative setting and for patients with respiratory illness. Though ubiquitous in high-income countries, safe pulse oximetry is still unavailable in many locations, including nearly 20% of operating theaters worldwide [1]. Pulse oximeters compute arterial hemoglobin oxygen saturation from the ratio of pulsatile to total transmitted red light divided by the same ratio for infrared light transilluminating the finger, ear, or other tissue. Theoretically, the derived saturation should remain independent of variables that remain constant throughout the cardiac cycle such as hemoglobin concentration, skin color, nail polish, and dirt. In practice, the accuracy of pulse oximeter measurements is influenced by a variety of factors including skin color, motion, variations in breathing, incorrectly applied probes, temperature, and low perfusion [2–7].

Accurate pulse oximeter measurements depend upon programming the oximeter with an empirically determined calibration. At present, calibration and validation of accurate instrument readings is accomplished via desaturation studies in human test subjects. In these studies, hypoxemia is induced in a stepwise fashion at a range of oxygen saturation levels from 100% down to approximately 70% so that photoplethysmographic data collected from the oximeter can be paired and referenced against gold standard CO-oximeter readings from collected arterial blood samples. In addition to being invasive and expensive, this calibration procedure is limited by the clinical and ethical ramifications of inducing hypoxemia below 70% in human subjects. Consequently, data need to be extrapolated for saturations below this, leading to potentially inaccurate readings at lower levels of oxygen saturation. More significantly, the vast majority of calibration and confirmation tests are done on healthy volunteer subjects and thus do not account for common clinical comorbidities such as severe anemia, low perfusion and motion.

Numerous studies, initially by Severinghaus et al., have demonstrated that pulse oximeter accuracy is severely compromised during profound hypoxemia [8, 9]. The effect of anemia on the accuracy of pulse oximetry at varying levels of oxygen saturation is less well understood, but greater measurement error has been demonstrated in subjects with low hematocrit levels [10]. Previous studies utilizing in vitro circulation models to investigate the performance of pulse oximeters during anemia [11] and dyshemoglobinemia [12] have suggested that the accuracy of a pulse oximeter may be dependent on hematocrit level. However, these studies are now decades old, and little has since been published about the accuracy of pulse oximeters during profound anemia and hypoxemia.

Although the presence of anemia is estimated at 9% in high-income countries, in low-income countries the prevalence is 43% [13]. In some settings in sub-Saharan Africa, 12–29% of hospitalized children are severely anemic (hemoglobin concentration less than 5.0 g per deciliter) [14]. Thus, if oximeter accuracy is impacted by severe anemia, then a significant proportion of patients may be at risk for inaccurate diagnosis and monitoring for hypoxemia. Furthermore, this likely disproportionately affects patients in low- and middle-income countries where anemia and lower-quality pulse oximeters are more common [15].

## Methods

**Fig. 1:**
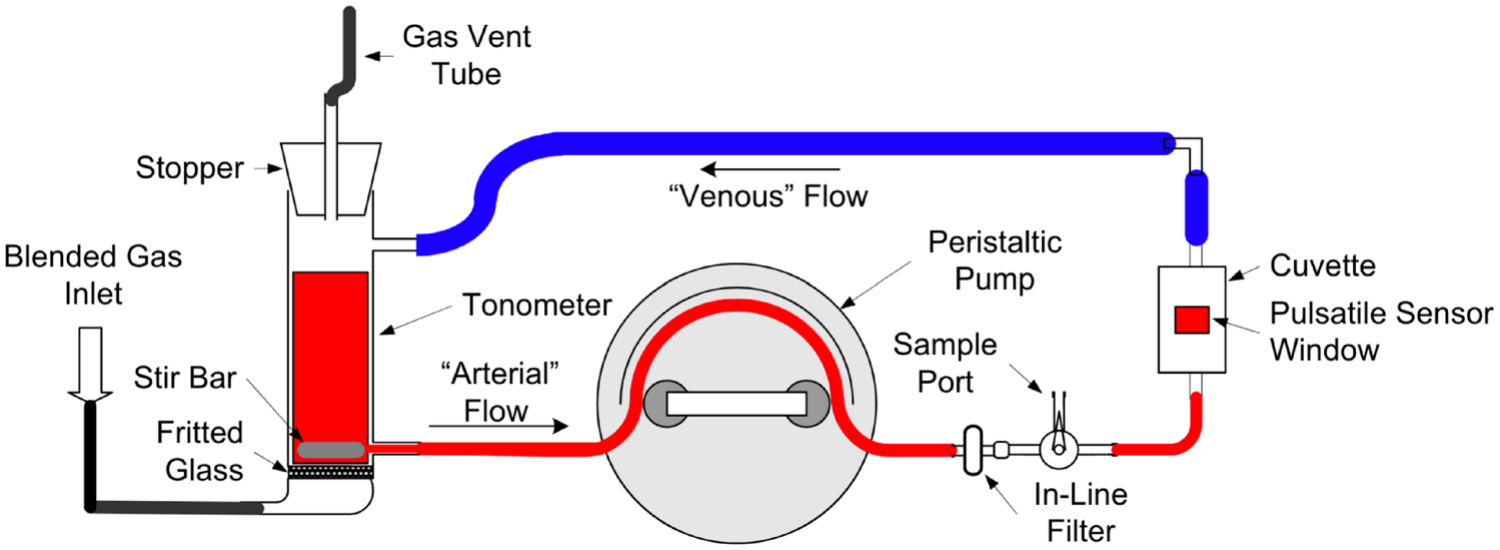
Simplified schematic of in vitro circulation system [16]

A custom in vitro circulation system using human blood was used for this protocol (Fig. 1). Fresh, single donor human whole blood in citrate-phosphate-dextrose (CPD) was obtained from a local community blood donation center. After centrifugation (3000 rpm for 10 min) and removal of the plasma, hemoconcentrated blood (Hct 79%) was mixed with normal saline to reach each of the four desired hematocrit levels (40%, 30%, 20%, and 10%), which were confirmed by HemoCue Hb 201+ and a Radiometer OSM3 CO-oximeter.

A custom tonometer was filled with 125 mL of whole blood at the desired hematocrit and thoroughly mixed. Oxygen and nitrogen were bubbled through fritted glass at the bottom of the tonometer and drops of antifoam were added until excessive foaming ceased. The blood was then pumped through the custom IVCS using a peristaltic pump set to generate a pulse rate of 65 BPM and gas flows adjusted until the desired fractional oxygen saturation (O_2_Hb) was achieved as measured by multi-wavelength oximetry using a reference invasive CO-oximeter (Radiometer OSM3, Copenhagen, Denmark).

Three oximeters were selected to reflect a range of cost. A benchtop Masimo Radical (S/N: 0666494) with Masimo LNCS Adtx sensor (Masimo Corporation, CA, USA), a handheld Acare AH-M1 (Acare Technology Co., Ltd., New Taipei City, Taiwan), and fingertip Contec CMS 50-DL (Contec Medical Systems Co., Ltd., Qinhuangdao, China). The Masimo Radical model used for data collection was an older model from approximately the early 2000s.

The emitter and detector from the three study pulse oximeters were applied to opposite sides of a custom pulsatile cuvette that was inline in the IVCS using adhesive. Care was taken to ensure no air spaces and perpendicular alignment was achieved through visual inspection and confirmed by device reading an SpO_2_. The pulse oximeters were connected to their respective processing unit. After ≥ 30 s of stability of SpO_2_ readings in a dark room, SpO_2_ and perfusion index readings from the pulse oximeter were recorded. A sample of blood was immediately withdrawn from a sample port adjacent to the cuvette and O_2_Hb measurement performed immediately on an OSM3. Nitrogen and oxygen were bubbled through the tonometer to reach a series of 6–10 stable target SpO_2_ plateaus between 60 and 100% (in 5–10% increments) and the above process was repeated for each plateau. The blood was mixed with normal saline to reach the next desired hematocrit level, reoxygenated to 100%, and the entire process was repeated for each hematocrit level. Proper emitter and sensor alignment on the cuvette was confirmed after all data were collected before switching to the next device. Carboxy-hemoglobin was monitored, and a new sample of blood was utilized if the CO-Hb level rose above 1.5%.

Light intensity dynamic range was limited in the CMS 50-DL, requiring alterations in the above protocol. At a hematocrit of 20 and O_2_Hb of 100%, the emitter flashed and turned off, indicating that likely too much light was reaching the detector. A neutral density filter was therefore placed between the emitter and cuvette to evenly reduce light transmission and bring light intensity within the dynamic range of the device. Optical density (OD) of the filter (0.5, 1.0, 1.5, 2, or 3) was selected by using the filter with the lowest OD (i.e. greatest transmission of light) that allowed for stable and accurate heart rate and SpO_2_ readings. A neutral density filter with OD 1.5 (3% transmission) was utilized for all oxygenation levels at hematocrit levels of 10 and 20. At a hematocrit of 30 with an OD 1.5 filter in place, the device failed to deliver readings due to insufficient light reaching the detector. With an OD 1.0 (10% transmission) filter in place, the device would provide readings with the room lights on but not with the room lights off, again suggesting insufficient light reaching the detector. With an OD 0.5 (32% transmission) filter in place, the device initially failed to provide readings due to too much light reaching the detector but functioned properly when the path length of the blood between emitter and detector was increased. At a hematocrit of 40, the OD 0.5 filter was kept in place and the path length was decreased back to normal. When the filter was removed completely at hematocrit 40, the device again failed to function due to too much light reaching the detector.

### Statistical analysis

The standard error of the estimate (SEE) was calculated for each pulse oximeter at a given hematocrit level and used to characterize pulse oximeter precision. Consistent with statistical methods used commonly by the US Food and Drug Administration (FDA) and International Organization for Standardization (ISO) which dictate the average root mean square error (A_RMS_) must be less than 3% throughout the O_2_Hb range 70–100%, we used A_RMS_ to characterize pulse oximeter accuracy and A_RMS_ greater than 3% as a threshold for inaccuracy. For the purposes of FDA certification, analysis requires greater than 200 data points. To extrapolate A_RMS_ calculations without the abundance of data points, a computed 2nd order equation was created at each hematocrit level for the three devices. The data were well fit to 2nd order equations as evidenced by low SEEs. For each device, the curve fit at hematocrit 40% was used as a “reference” performance level and was subtracted from the curve fits at hematocrits of 30%, 20%, and 10%. The bias (%) and A_RMS_ (%) were calculated over the 70–100% O_2_Hb range and at each 10% O_2_Hb range down to 60% per ISO criteria.

## Results

### Accuracy of hematocrit data

The hematocrit of the experimented blood used in the in vitro calibration circuit was confirmed using the Radiometer OSM3 CO-oximeter for each pulse oximeter device at the desired hematocrit level (Table 1). The hematocrit levels were estimated from the Total Hemoglobin (tHb) values measured on the OSM3 using the relationship Hct = 3*tHb. For all devices, the hematocrit levels obtained for each test were close to the desired nominal hematocrit (mean Hct ≤ 0.5%; StDev ≤ 0.42%) and stable throughout testing.

**Table 1:** Hematocrit levels tested for all oximeters

Device	Nominal Hct level	N	Hct (%) Mean	Hct (%) StDev	Perfusion Index (%) Mean ± SD
Masimo Radical	40	6	39.2	0.20	8.1 ± 0.4
	30	7	30.5	0.17	6.7 ± 1.2
	20	7	20.5	0.13	5.4 ± 0.5
	10	9	10.1	0.18	3.4 ± 1.5
Acare AH-M1	40	8	40.0	0.30	–
	30	9	30.3	0.09	–
	20	8	19.7	0.05	–
	10	7	10.0	0.16	–
CMS 50-DL	40	8	40.5	0.42	–
	30	8	29.9	0.23	–
	20	9	19.9	0.30	–
	10	10	10.4	0.28	–

Hematocrit levels were estimated from the Total Hemoglobin (tHb) values measured on the Reference CO-oximeter using the relationship Hct ≈ 3*tHb. Perfusion Index (PI) was only provided for the Masimo radical. The Acare AH-M1 and CMS devices do not measure PI

### Perfusion index at hematocrit levels

At lower hematocrits, the perfusion index decreased as measured by the Masimo Radical device (Table 1). This perfusion index data was only available on the Masimo Radical device.

### Precision of pulse oximeters

All pulse oximeters tested showed varying degrees of precision in reporting the SpO_2_ from the IVCS system with increased variability observed at lower hematocrits (Table 2). The Masimo pulse oximeter had the lowest SEEs (SEE 0.35–0.66%) among the three tested devices, even at the lowest tested hematocrit level. The Acare AH-M1 pulse oximeter performed similarly to the Masimo device at hematocrit levels greater than 10 (SEE 0.4–0.51); however, at a hematocrit of 10, the Acare AH-M1 had the highest SEE for any device at any hematocrit level. The CMS 50-DL had the greatest variability in precision throughout all hematocrit levels tested (SEE 0.94–1.26), and this variability did not correlate with worsening anemia.

**Table 2:** IVCS coefficients and standard error of the estimate (SEE) for polynomial curve fits for the three oximeters at each hematocrit level

Device	Hct level	Coefficient “C2”	Coefficient “C1”	Coefficient “B”	SEE (%)
Masimo radical	40	− 3.11E−03	1.29E+00	3.50E+00	0.35
	30	− 3.26E−03	1.31E+00	3.07E+00	0.36
	20	− 3.31E−03	1.34E+00	1.03E+00	0.66
	10	1.93E−03	3.93E−01	4.42E+01	0.44
Acare AH-M1	40	− 9.91E−03	2.44E+00	− 4.48E+01	0.51
	30	− 7.31E−03	2.06E+00	− 3.20E+01	0.40
	20	− 8.79E−03	2.36E+00	− 4.60E+01	0.40
	10	− 5.42E−03	1.95E+00	− 3.81E+01	3.74
CMS 50-DL	40	2.98E−03	3.34E−01	3.84E+01	1.26
	30	− 1.50E−03	1.04E+00	1.16E+01	0.94
	20	2.56E−03	3.40E−01	4.24E+01	1.20
	10	1.64E−03	3.69E−01	4.69E+01	1.00

Higher SEE values indicate greater variability and thus decreased precision

### Accuracy of pulse oximeters and concordance data at normocythemia

**Fig. 2:**
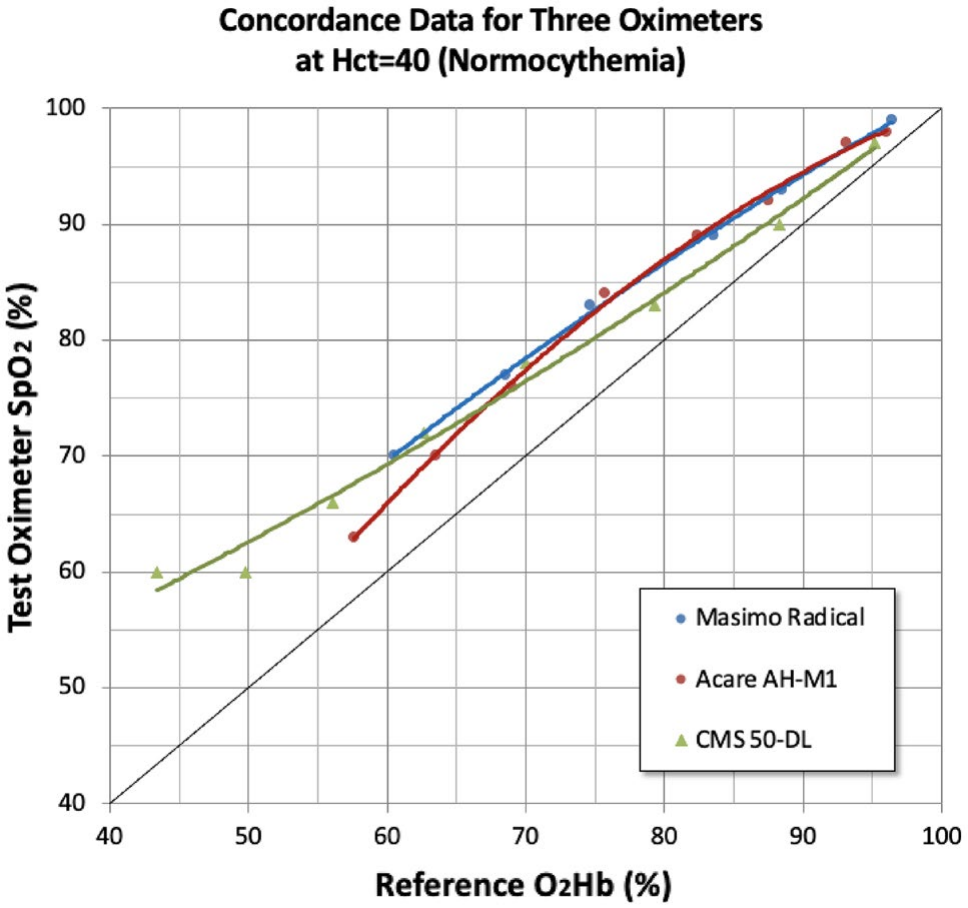
IVCS concordance data for three oximeters at Hct = 40 (normocythemia). The ability of pulse oximeters to accurately measure SpO_2_ in an in vitro system was compared to the human calibration data at normocythemia (Hct = 40%). In vitro measurements on IVCS do not exactly match invasive measurements on a reference device, indicated by deviation from the line of identity. Note however, the concordance of the benchtop and handheld oximeters compared to the fingertip device

The ability of pulse oximeters to accurately measure SpO_2_ in an in vitro system was compared to the human calibration data at normocythemia (Hct = 40%) (Fig. 2). The comparison of test oximeter SpO_2_ (%) to human reference O_2_Hb (%) shows an expected error between human and IVCS calibrations. This represents a well-known and previously documented difference that exists between human and IVCS calibration of pulse oximeters [17]. Above 75% O_2_Hb, the Masimo and Acare devices reported nearly identical SpO_2_ values on human subjects, as seen with the overlapping regression curves. For the CMS 50-DL device, the oximeter SpO_2_ neither matched the Masimo and Acare readings, nor did the error between IVCS and human calibrations remain constant at varying O_2_Hb.

### Accuracy of the in vitro calibration to measure SpO_2_ with varying hematocrits

Acknowledging the expected calibration error between in vitro and human calibration, it was possible to assess the accuracy of the in vitro calibration at different hematocrit levels. The calibration errors measured at normocythemia (Hct ~ 40%) were zeroed, thus any change in SpO_2_ measurement was assumed to be due to changes in hematocrit.

**Fig. 3:**
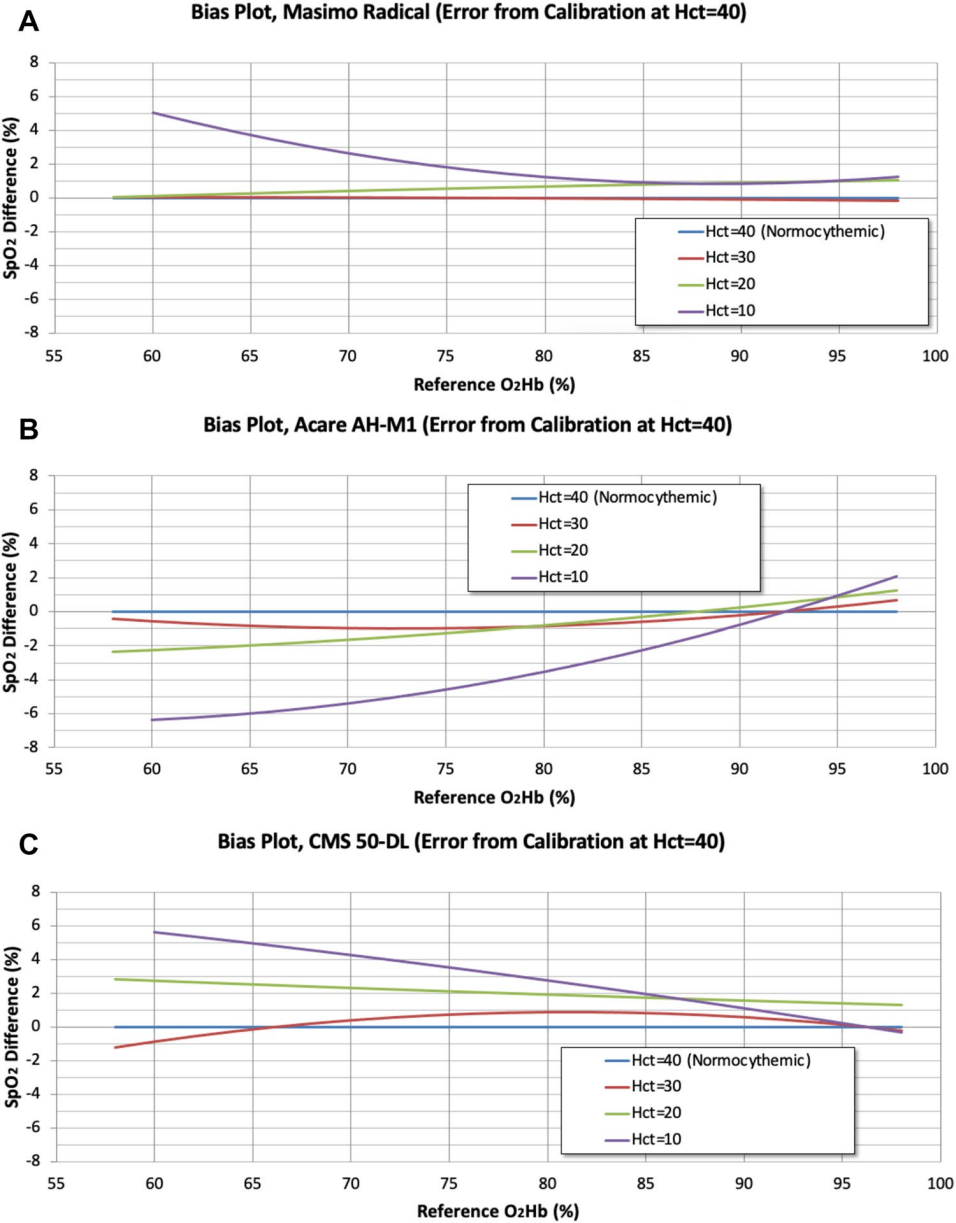
**a–c** Bias plots showing errors in SpO_2_ measurement as a function of reference oxygen saturation (O_2_Hb) and hematocrit (Hct). Errors shown are the differences in saturation measurements from those obtained for Hct = 40; therefore, by definition, the zero-error line is the Hct = 40 line. To obtain the curves shown in these plots, the curve fit for Hct = 40 was subtracted from the curve fit at each of the other hematocrit levels. The Hct = 10 and 20 curves for the CMS 50-DL oximeter were truncated below 58% O_2_Hb

As seen in Fig. 3a–c, the accuracy of SpO_2_ decreases as the hematocrit falls for all devices and is more pronounced at lower O_2_Hb measurements. An SpO_2_ error greater than 3% was used as a threshold for inaccuracy. The Masimo device had nearly identical SpO_2_ measurements at Hct levels of 20, 30, and 40 regardless of O_2_Hb; however, at Hct of 10, the SpO_2_ error was greater than 3% when O_2_Hb values dropped below 68%. The Acare device was not as precise as the Masimo for hematocrit levels of 20 and above, at a hematocrit of 10 the SpO_2_ error was greater than 3% for O_2_Hb values below 82%. Of note, this device had the highest A_RMS_ of those tested (~ 6%) for hematocrit levels of 10 below O_2_Hb of 60%, suggesting this device is most inaccurate for low hematocrits and low oxygen states. The CMS 50-DL device showed the greatest inaccuracy as a function of hematocrit. These inaccuracies occurred at a higher hematocrit level than the other devices (Hct 20). At a hematocrit of 10, the SpO_2_ error greater than 3% occurred at O_2_Hb values of 78%.

### Accuracy of in vitro calibration results using bias and precision

The Masimo device (Table 3) had an A_RMS_ less than 3% for all hematocrits tested between 70 and 100%, which is the A_RMS_ threshold established by the ISO for in vivo performance testing. This device had an A_RMS_ greater than 3% for severe anemia (Hct 10%) at O_2_Hb range 60–69.9% (Bias 3.76%, A_RMS_ 3.83%). The Acare device (Table 4) performed well with hematocrit levels greater than 10%, but with greater bias and A_RMS_ than the Masimo device. At a hematocrit of 10%, the Acare was not accurate at O_2_Hb values between 60–69.9% (Bias − 5.97%, A_RMS_ 5.98%), 70–79.9% (Bias − 4.56%, A_RMS_ 4.59%), and 70–100% (Bias − 1.93%, A_RMS_ 3.10%). The CMS 50-DL (Table 5) showed greater bias and A_RMS_ than the two other devices starting at a hematocrit 20%, despite having an A_RMS_ of less than 3% at each O_2_Hb range. At a hematocrit of 10%, the CMS 50-DL was similarly not accurate at O_2_Hb ranges less than 79.9% (Fig. 3c).

**Table 3:** Masimo radical SpO_2_ bias and A_RMS_ statistics for differences from SpO_2_ at Hct = 40%

O_2_Hb range	HCT = 30		HCT = 20		HCT = 10	
	Bias (%)	A_RMS_ (%)	Bias (%)	A_RMS_ (%)	Bias (%)	A_RMS_ (%)
60–69.9	0.04	0.04	0.27	0.28	3.76	3.83
70–79.9	0.01	0.02	0.54	0.54	1.85	1.89
80–89.9	− 0.06	0.06	0.77	0.78	0.95	0.95
90–100	− 0.15	0.15	0.97	0.97	1.05	1.07
70–100	− 0.07	0.10	0.76	0.78	1.28	1.37

For each device, the curve fit at hematocrit 40% was used as a “reference” performance level and was subtracted from the curve fits at hematocrits of 30%, 20%, and 10%. The bias (%) and A_RMS_ (%) were calculated over the 70–100% O_2_Hb range and at each 10% O_2_Hb range down to 60%. A_RMS_ is used to characterize pulse oximeter accuracy and A_RMS_ greater than 3% used as a threshold for inaccuracy

**Table 4:** Acare AH-M1 SpO_2_ bias and A_RMS_ statistics for differences from SpO_2_ at Hct = 40%

O_2_Hb range	HCT = 30		HCT = 20		HCT = 10	
	Bias (%)	A_RMS_ (%)	Bias (%)	A_RMS_ (%)	Bias (%)	A_RMS_ (%)
60–69.9	− 0.82	0.83	− 1.96	1.97	− 5.97	5.98
70–79.9	− 0.97	0.97	− 1.24	1.26	− 4.56	4.59
80–89.9	− 0.59	0.62	− 0.29	0.42	− 2.25	2.39
90–100	0.31	0.46	0.88	0.96	0.97	1.45
70–100	− 0.41	0.71	− 0.21	0.95	− 1.93	3.10

For each device, the curve fit at hematocrit 40% was used as a “reference” performance level and was subtracted from the curve fits at hematocrits of 30%, 20%, and 10%. The bias (%) and A_RMS_ (%) were calculated over the 70–100% O_2_Hb range and at each 10% O_2_Hb range down to 60%. A_RMS_ is used to characterize pulse oximeter accuracy and A_RMS_ greater than 3% used as a threshold for inaccuracy

**Table 5:** CMS 50-DL SpO_2_ bias and A_RMS_ statistics for differences from SpO_2_ at Hct = 40%

O_2_Hb range	HCT = 30		HCT = 20		HCT = 10	
	Bias (%)	A_RMS_ (%)	Bias (%)	A_RMS_ (%)	Bias (%)	A_RMS_ (%)
60–69.9	− 0.18	0.41	2.52	2.52	4.93	4.94
70–79.9	0.69	0.70	2.10	2.10	3.50	3.53
80–89.9	0.79	0.79	1.72	1.72	1.92	1.98
90–100	0.10	0.33	1.37	1.37	0.18	0.56
70–100	0.52	0.64	1.73	1.76	1.86	2.35

For each device, the curve fit at hematocrit 40% was used as a “reference” performance level and was subtracted from the curve fits at hematocrits of 30%, 20%, and 10%. The bias (%) and A_RMS_ (%) were calculated over the 70–100% O_2_Hb range and at each 10% OHb range down to 60%. A_RMS_ is used to characterize pulse oximeter accuracy and A_RMS_ g_2_reater than 3% used as a threshold for inaccuracy

## Discussion

In vivo validation studies, currently the standard for pulse oximeter performance validation, are expensive, time consuming and with significant limitations including the inability to study performance during severe anemia. This study is the first to use an in vitro circulation system (designed and built by Kestrel Labs, Inc. and described elsewhere [16]) to demonstrate degradation of oximeter performance during severe anemia for three commercial pulse oximeters of varying cost.

There are relatively few prior studies investigating the impact of severe anemia on pulse oximeter accuracy. Severinghaus and Koh reported increased error in anemic humans when SaO_2_ dropped below 75% [10]. Lee et al. utilized a canine model to find pulse oximetry underestimated SaO_2_ by 5.4% ± 18.8% with hematocrit diluted below 10% [19]. On the contrary, in a case series including 17 patients with acute severe anemia due to hemorrhage, Jay et al. found pulse oximetry to be accurate and reliable at a hemoglobin concentration of 2.3 g/dL in the absence of hypoxia [20]. Similarly, Perkins et al. studied the effects of anemia and acidosis on pulse oximeter bias in the critically ill and found SpO_2_ underestimates SaO_2_ to a greater extent with progressive anemia, though the clinical significance of the findings was small in the absence of hypoxia [27]. Overall, prior work has demonstrated that the impact of anemia on SpO_2_ measurements in normoxic individuals is small and likely becomes clinically relevant only when severe anemia is combined with hypoxia with an underestimation of SaO_2_ under these conditions. This underestimation has been described as fortuitous since it overestimates the degree of desaturation in anemic subjects where harm is potentially the greatest and earlier detection of hypoxemia may lead to earlier administration of supplemental oxygen.

Notably, all oximeters we tested showed loss of accuracy with decreasing hematocrit but these error trends did not occur in the same direction for all three oximeters. In contrast to the underestimation of SaO_2_ described in previous literature, our work suggests that pulse oximeter design may play a role in determining the direction of bias during severe anemia and hypoxia. Further investigation with additional oximeters would elucidate this observation.

In our study, the most expensive device tested (Masimo Radical) maintained good accuracy at all but the most extreme anemic hematocrit level. The intermediate cost device (Acare AH-M1) was not as accurate as the Masimo, and inaccuracies appeared at relatively higher levels when compared to the Masimo device. The least expensive device studied (CMS 50-DL) was the least accurate with the poorest dynamic range of the three oximeters. Prior studies including the Acare AH-M1 [21] and Contec CMS-50DL [15] have shown these devices meet standardized criteria for accuracy in healthy volunteers; however, we are not aware of any data investigating the performance of any of these devices in subjects with severe anemia.

Currently there are a small number of in vitro calibration devices that have been reported and few that are commercially available. Attempts to develop an in vitro calibration system date back to as early as the 1990s. Reynolds et al. developed an in vitro test system to study the accuracy of ten different oximeters at low oxyhaemoglobin saturations [17]. De Kock and Tarassenko developed an in vitro blood circuit with a flexible cuvette to investigate theoretical models of optical transmission in whole blood [22]. Hornberg et al. developed a novel pulse oximeter calibration technique utilizing a spectral light modulator as a calibration standard [23].

Commercially available devices (including the Fluke Biomedical ProSim 8 [24] or SPOT Light SpO_2_ Functional Tester [25] and WhaleTeq AECG100 [18]), are frequently misunderstood and potentially inappropriately utilized by researchers hoping to quickly ‘validate’ the performance on an oximeter. While these devices do play an important role in device development and performance verification, existing devices are intended to validate performance for devices that have calibration curves pre-programmed into the testing device. They provide an optical signal to verify that the electronics within the pulse oximeter probe are functional during preventative maintenance checks on patient monitors in service. In other words, if the oximeter is known to the testing device then the testing device can assess if the oximeter performs against a simulated signal in an expected way. According to manufacturer documentation, they are not intended to be used to calibrate medical equipment. They should not be used to assess performance of pulse oximeters unknown to the testing device. No commercially available devices use real blood. Current work is underway to better characterize and improve the utility of commercially available in vitro devices.

The study had several limitations, the most significant of which is the performance of the IVCS device. In comparison to previously published IVCS devices, the IVCS used in the current study proved to be the most accurate to date [11, 26]. Nonetheless, performance did not equivalently reproduce in vivo performance. Thus, a zeroing factor was utilized for our analysis, and as was the case for the least expensive oximeter (CMS 50-DL), we had to place a neutral density filter in the optical path for the device to produce a result. The Acare unit displayed the correct heart rate, but the audible pulse indicator worked approximately every other beat. The Masimo Radical used in the study was from the early 2000s and may not be representative of the latest Masimo technology. The applicability of our IVCS findings to real world performance of these pulse oximeters in clinical settings is unclear.

Future work is needed to continue to refine the performance of IVCS to eventually produce a system that more precisely mimics in vivo performance. An additional limitation of the study was the necessary deconstruction of the oximeter probes for attachment to the IVCS. Probe positioning and design is an important real-world factor in oximeter performance which we could not completely account for in this study. Although we studied three devices spanning a range of cost, our study was not a comprehensive analysis of how anemia affects pulse oximeter accuracy in a wide range of pulse oximeter types, models, and probe configurations.

## Conclusion

Pulse oximeter performance is impacted by severe anemia in vitro, though applicability of these findings to clinical performance for these devices is uncertain. The development of in vitro calibration systems to evaluate pulse oximeter performance can play a role in understanding and improving pulse oximeter performance because they allow testing in a more controlled manner and over wider ranges of physiologic conditions than in vivo studies. This study presents an IVCS device that was able to report SpO_2_ levels during states of extreme anemia and hypoxemia. Further studies are warranted to fully characterize the impact of anemia on oximeter performance, including further development of better in vitro circulation systems as well as in vivo studies in the clinical setting. In vitro devices may play a role in augmenting in vivo performance studies required for FDA and ISO certification.
